# Coronary heart disease mortality in treated familial hypercholesterolaemia: Update of the UK Simon Broome FH register

**DOI:** 10.1016/j.atherosclerosis.2018.04.040

**Published:** 2018-07

**Authors:** S.E. Humphries, J.A. Cooper, M. Seed, N. Capps, P.N. Durrington, B. Jones, I.F.W. McDowell, H. Soran, H.A.W. Neil, the Simon Broome Familial Hyperlipidaemia Register Group

**Affiliations:** a Centre for Cardiovascular Genetics, Institute of Cardiovascular Science, University College London, University Street, London, WC1E 6JJ, UK; b Department of Cardiology, Imperial College Faculty of Medicine, Charing Cross Campus, University of London, UK; c Department of Clinical Biochemistry, The Shrewsbury and Telford Hospital NHS Trust, Princess Royal Hospital, Telford, UK; d Cardiovascular Research Group, School of Clinical and Laboratory Sciences, University of Manchester, UK; e Section of Investigative Medicine, Imperial College London, UK; f Department of Medical Biochemistry and Immunology, University Hospital of Wales, Cardiff, UK; g Central Manchester University Hospitals NHS Foundation Trust, Manchester, UK; h Wolfson College, University of Oxford, UK

**Keywords:** Heterozygous familial hypercholesterolemia, Coronary mortality, Cancer mortality, Dutch lipid clinic network score

## Abstract

**Background and aims:**

Patients with familial hypercholesterolaemia (FH) have an elevated risk of coronary heart disease (CHD). Here we compare changes in CHD mortality in patients with heterozygous (FH) pre 1992, before lipid-lowering therapy with statins was used routinely, and in the periods 1992–2008 and 2008–2016.

**Methods:**

1903 Definite (DFH) and 1650 Possible (PFH) patients (51% women) aged 20–79 years, recruited from 21 lipid clinics in the United Kingdom and followed prospectively between 1980 and 2016 for 67,060 person-years. The CHD standardised mortality ratio (SMR) compared to the population in England and Wales was calculated (with 95% Confidence intervals).

**Results:**

There were 585 deaths, including 252 from CHD. Overall, the observed 2.4-fold excess coronary mortality for treated DFH post-1991 was significantly higher than the 1.78 excess for PFH (35% 95% CI 3%–76%). In patients with DFH and established coronary disease, there was a significant excess coronary mortality in all time periods, but in men it was reduced from a 4.83-fold excess (2.32–8.89) pre-1992 to 4.66 (3.46–6.14) in 1992–2008 and 2.51 (1.01–5.17) post-2008, while in women the corresponding values were 7.23 (2.65–15.73), 4.42 (2.70–6.82) and 6.34 (2.06–14.81). Primary prevention in men with DFH resulted in a progressive reduction in coronary mortality over the three time-periods, with no excess mortality evident post-2008 (0.89 (0.29–2.08)), although in women the excess persisted (post-2008 3.65 (1.75–6.72)).

**Conclusions:**

The results confirm the benefit of statin treatment in reducing CHD mortality, but suggest that FH patients with pre-existing CHD and women with FH may not be treated adequately.

## Highlights

• 1903 Definite (DFH) and 1650 Possible (PFH) patients followed between 1980 and 2016.
• Post 1992 treated DFH patients had 35% higher coronary mortality than PFH patients.
• In DFH men without CHD, coronary mortality was not significantly elevated post 2008.
• In DFH women coronary mortality was significantly elevated at all times.
• Suggests that, compared with men, women with FH may not be being treated adequately.

## 1 Introduction

Familial hypercholesterolemia (FH) is a common autosomal dominant disorder with the frequency of heterozygous FH being estimated at 1 in 200–500 in most European populations [[1], [2], [3]]. Patients with FH are exposed to elevated low density lipoprotein cholesterol (LDL-C) concentrations from birth and are at a higher risk of Coronary Heart Disease (CHD) for any given LDL-C concentration [4]. In the heterozygous condition the cumulative risk of CHD by the age of 60 years without effective treatment is at least 50% in men and about 30% in women [[5], [6], [7]]. Before effective treatment with lipid-lowering therapies such as statins became available, mortality from CHD was increased nearly 100-fold in young adults aged 20–39 years, and about 4-fold for patients aged 40–59 years, but for those surviving through middle age, risk was similar to the rates of CHD in the general population of England and Wales [8,9].

In the UK, the Simon Broome FH register criteria [1,10] are used to classify patients clinically into Definite FH (DFH) or Possible FH (PFH) [1,10]. A diagnosis of DFH is made if the patient has low density lipoprotein-cholesterol (LDL-C) level >4.9 mmol/l and tendon xanthoma (TX), while a diagnosis of PFH is made if the patient has elevated cholesterol levels and a family history of hypercholesterolemia or early CHD. A diagnostic scoring tool has also been developed by the Dutch Lipid Clinic Network (DLCN) where each subject is given increasing points for increasing levels of untreated LDL-C, having TX and a family history of early CHD [10]. Those with a score >8 have Definite FH, those with a score between 5 and 8 have probable FH, 3–5 possible FH and a score below 3 are designated as not-FH.

Mutations in three genes, the LDL-receptor gene (*LDLR*), the gene coding for apolipoprotein B (*APOB*) and the gene encoding protein convertase subtilisin/kexin 9 (*PCSK9*), are known to cause FH [11]. In UK FH patients where a monogenic cause can be found, approximately 93% carry a mutation in the *LDLR* gene, 5% in *APOB* and 2% in *PCSK9* [11]. Although other novel genes have been proposed [12], none have yet been independently confirmed. While in DFH patients an FH-causing mutation can be identified in about 80% of subjects, this falls to only 20–30% in PFH patients [13]. In the majority of patients with a clinical diagnosis of FH but with no detectable mutation in any of the three common genes [14], it is now widely accepted that there is a polygenic cause for their raised LDL-C level [15,16].

Previous results from the UK Simon Broome Register have shown that since the introduction and widespread use of statins, the prognosis for patients with heterozygous FH has improved markedly, with a reduction in coronary mortality of about a third. Importantly, in patients with no known CHD at registration (i.e., primary prevention), all-cause mortality in treated FH patients is significantly lower than in the general population, mainly due to a reduction of more than a third in the risk of fatal cancer. This was probably because of the lower prevalence of cigarette smoking in FH patients than in the general population [9,17], due to adherence to advice to be physically active, make dietary changes, avoid obesity and not to smoke cigarettes. The aim of this paper was to extend our previous reports [9,17,18] by studying an enlarged cohort of 3553 heterozygous patients followed for up to 36 years until the end of 2016, by when the exposure had increased to 67,760 person-years. This has allowed us to examine the changes in mortality compared with the general population of England and Wales (Standardised Mortality Ratio, SMR) both before and after the routine use of statins.

## 2 Materials and methods

The methods have been described previously [17]. The characteristics of patients at registration were recorded on a standard registration form. A fasting venous blood specimen taken at the registration visit was used to determine serum total cholesterol, triglycerides, and high density lipoprotein, and was measured by the laboratories routinely used by the participating clinics. LDL-C concentrations were calculated using the Friedewald formula [19]. Registered patients were flagged by the National Health Service Central Registry and, in the event of death, a copy of the death certificate was provided. The underlying cause of death was coded by one investigator using the International Classification of Disease (ICD) 9th revision. All patients gave informed consent for inclusion in the Register. The study received approval from the local ethics committee of each participating centre. Patients were classified as having either Simon Broome (SB) Definite FH or Possible FH using published criteria [10,17], or by using the Dutch Lipid Clinic Network as described [10] and as shown in Supplementary methods, with those with a score >8 having Definite FH, those with a score between 5 and 8 Probable FH, with a score 3–5 as Possible FH and those with a score below 3 designated as not-FH. Since the majority of the subjects have not had DNA tests, this component was not included in the score.

The analysis used a standard computer program for cohort studies [20]. Person-years of risk were aggregated into 5-year age groups and 5-year calendar periods and the expected number of deaths from specified causes were estimated. A total of 571 subjects were censored on reaching the age 80 years, and a further 50 patients who had emigrated were censored at the date of embarkation. The expected number of deaths from CHD (ICD codes 4100–4149); stroke (4300–4389); non-coronary causes (10–4099 & 415–9999); cancers (1400–2089); site specific cancers, accidents and violence (8000–9999); and total mortality were calculated by applying the age and calendar-specific death rates for men and women in the general population of England and Wales to the person years accumulated by men and women in the cohort. The standardised mortality ratio (SMR) was calculated from the ratio of the number of deaths observed to those expected, and was expressed as a percentage (SMR = 100 for the reference population), and the exact 95% confidence intervals were calculated. The test of significance used was a two-sided Poisson probability of observing the number of deaths that occurred given the expected number of deaths. A Cox-proportional hazard model was used to identify univariate baseline characteristics that were significantly associated with CHD mortality, and a stepwise model used to identify those that were independently associated using a significance level of 0.05 for entry to the model and 0.10 for elimination. A term for DFH/PFH was forced into the model to examine whether the higher risk in DFH patients was explained by these factors.

## 3 Results

A total of 3557 patients were registered and followed up between 1 January 1980 and 31 December 2015. We excluded 2 patients whose vital status was unknown and 2 patients aged more than 80 years at registration. The resulting cohort of 3553 patients was followed for 67,760 person years with a median duration of follow up of 20 years. As shown in Supplementary Table 1 it consisted of 1724 Male and 1829 female patients, and as shown in Supplementary Table 2, of these 1903 had a diagnosis of SB DFH and 1650 of PFH. At registration, 27% of men and 18.7% of women had known CHD (Supplementary Table 1) defined as either a history of a previous myocardial infarction, angina, a coronary artery by-pass graft, or angioplasty, but history of CHD was not significantly different between DFH and PFH patients (Supplementary Table 2). As expected, those with DFH had significantly higher total and LDL-C and lower HDL-C than PFH patients who had higher systolic and diastolic blood pressure, a higher rate of smoking and BMI, and a higher prevalence of type 2 diabetes. Before treatment the mean total cholesterol concentration was on average 0.68 mmol/l and LDL-C 1.01 mmol/l higher in DFH compared to PFH subjects (both p < 0.001).

Table 1 shows the observed and expected number of deaths by major cause and time period. In total, there were 585 deaths from all causes and 252 from CHD. As shown in Table 2, within the FH sample studied risk was independently related to age, sex, smoking, previous CHD, total cholesterol and DFH/PFH. Overall, the hazard ratio for DFH vs PFH is 1.37 (1.07–1.77) *p*=0.013 before adjustment and 1.21 (0.93–1.58) *p*=0.162 after adjustment for all variables in the stepwise model. The ten year CHD mortality rate (Kaplan-Meier estimate) for those without CHD at baseline is 1.2% and for those with prior CHD it is 10.7%. After adjusting for age this equates to a rate of 0.8% at age 40, 1.4% at age 50 and 2.6% at age 60 for those without prior CHD, with rates in those with prior CHD being 7.2%, 9.4% and 12.4% respectively.

**Table 1:** Observed and expected deaths by major cause and time period in DFH + PFH patients all ages.

	From 1 January 1980 to 31 December 1991 Person-years exposure = 6627 years					From 1 January 1992 to December 2008 Person-years exposure = 43,117 years				
	Observed	Expected	SMR	95% CI	*p*-value	Observed	Expected	SMR	95% CI	*p*-value
Coronary heart disease	36	11.30	319	(223, 441)	<0.0001	162	79.40	204	(174, 238)	<0.0001
Stroke	1	2.91	34	(1, 192)	0.43	21	27.73	76	(47, 116)	0.23
Accidents & violence	1	2.22	45	(1, 251)	0.70	6	9.98	60	(22, 131)	0.26
All cancers	14	14.71	95	(52, 160)	0.99	91	140.53	65	(52, 80)	<0.0001
All-causes of death	54	40.49	133	(100, 174)	0.05	356	385.05	92	(83, 103)	0.14

	From 1 January 2009 to 31 December 2015 Person-years exposure = 17,317 years					*p*-value trend
	Observed	Expected	SMR	95% CI	*p*-value	
Coronary heart disease	54	23.23	232	(175, 303)	<0.0001	0.31
Stroke	6	9.19	65	(24, 142)	0.38	0.77
Accidents & violence	1	2.47	40	(1, 225)	0.58	1.00
All cancers	56	60.69	92	(70, 120)	0.60	0.26
All-causes of death	175	153.08	114	(98, 133)	0.09	0.61

**Table 2:** Univariate and multivariate factors associated with CHD mortality in DFH plus PFH patients combined.

Variable		Univariate associations with CHD death		Multivariable model including all variables		Model selected using stepwise Cox regression	
		HR (95% CI)	*p* value	HR (95% CI)	*p* value	HR (95% CI)	*p* value
Age (years)	Per 10years	1.87 (1.57–2.21)	<0.0001	1.49 (1.21–1.83)	0.0001	1.52 (1.26–1.82)	<0.0001
Sex	F:M	0.45 (0.34–0.58)	<0.0001	0.48 (0.35–0.65)	<0.0001	0.49 (0.37–0.64)	<0.0001
Ever smoker	Y:N	1.96 (1.51–2.54)	<0.0001	1.70 (1.26–2.30)	0.0005	1.60 (1.22–2.10)	0.001
Diabetes	Y:N	2.03 (1.00–4.10)	0.05	1.91 (0.70–5.19)	0.207		
Total cholesterol (mmol/l)	Per SD	1.37 (1.23–1.52)	<0.0001	1.39 (1.24–1.56)	<0.0001	1.40 (1.26–1.55)	<0.0001
BMI (kg/m^2^)	Per SD	1.12 (0.97–1.30)	0.128	1.08 (0.89–1.30)	0.441		
SBP (mmHg)	Per SD	1.09 (0.96–1.25)	0.198	1.07 (0.92–1.25)	0.381		
Prior CHD	Y:N	5.76 (4.38–7.57)	<0.0001	4.19 (3.05–5.74)	<0.0001	4.33 (3.24–5.80)	<0.0001
Diagnosis	DFH:PFH	1.37 (1.07–1.77)	*p* = 0.013	1.32 (0.98–1.77)	0.072		

Separate analyses for mortality were undertaken for the time period before January 1992, when statins were not routinely used, between January 1992–December 2008, during which time statin treatment became widely available, and from 2009 to December 2015, when it would be expected that FH patients would have their LDL-C levels managed with high potency statin treatment and or combination therapy with other lipid lowering agents such as ezetimibe. As shown in Table 1, there was significant excess coronary mortality for all three periods, falling a 3.19-fold excess (2.23–4.41) to 2.04 (1.74–2.38) to 2.32 (1.75–3.03). There was a significant excess mortality from all causes before, but not after, 1 January 1992, due mainly to a lower SMR for cancer.

We next compared the CHD SMR (95% CI) in statin treated Simon Broome defined DFH and PFH. As shown in Table 3, the CHD SMR was higher at all times in DFH compared to PFH patients, and using the combined data post 1991 for 1903 DFH patients (149 events in 36,625 person years follow-up) compared to1650 PFH patients (103 events, 30,435 person years follow-up), the 2.4-fold excess coronary mortality in treated DFH was significantly higher than the 1.78 excess in PFH (35%, 95% CI 3–76%, *p* = 0.03)). We also compared the CHD SMR using the Simon Broome and DLCN score system. As shown in Supplementary Fig. 1, there was no significant difference in the excess coronary mortality between SB DFH patients and DLCN Definite FH (ie score >8) (2.53 (2.14–2.97) *vs*. 2.53 (2.07–2.05)) and in PFH and DLCN <8 (1.85 (1.51–2.24) *vs*. 1.65 (1.30–2.06)).

**Table 3:** Observed and expected CHD and Total deaths by time period in DFH vs PFH patients (all ages).

	Total Pyrs	From 1 January 1980 to 31 December 1991					Total Pyrs	From 1 January 1992 to December 2008				
		Observed	Expected	SMR	95% CI	*p*-value		Observed	Expected	SMR	95% CI	*p*-value
**DFH patients**												
Coronary heart disease	3887	24	6.72	357	(229, 531)	<0.0001	23,278	98	39.96	245	(199, 299)	<0.0001
All cause of death	3887	37	23.69	156	(110, 215)	0.01	23,278	194	192.77	100	(87,116)	0.95
**PFH patients**												
Coronary heart disease	2740	12	4.58	262	(135, 458)	0.0005	19,839	64	39.44	162	(125, 207)	0.0004
All-causes of death	2740	17	16.80	101	(59, 162)	1.00	19,839	162	192.28	84	(72, 98)	0.03

	Total Pyrs^a^	From 1 January 2009 to 31 December 2015					*p*-value trend
		Observed	Expected	SMR	95% CI	*p*-value	
**DFH patients**							
Coronary heart disease	9460	27	11.92	227	(149, 330)	0.0002	0.16
All-causes of death	9460	84	78.66	107	(85, 132)	0.0.58	0.25
**PFH patients**							
Coronary heart disease	7857	27	11.31	239	(157, 347)	0.0001	0.70
All-causes of death	7857	91	74.42	122	(98, 150)	0.07	0.02

a Pys = person years exposure.

**Fig. 1:**
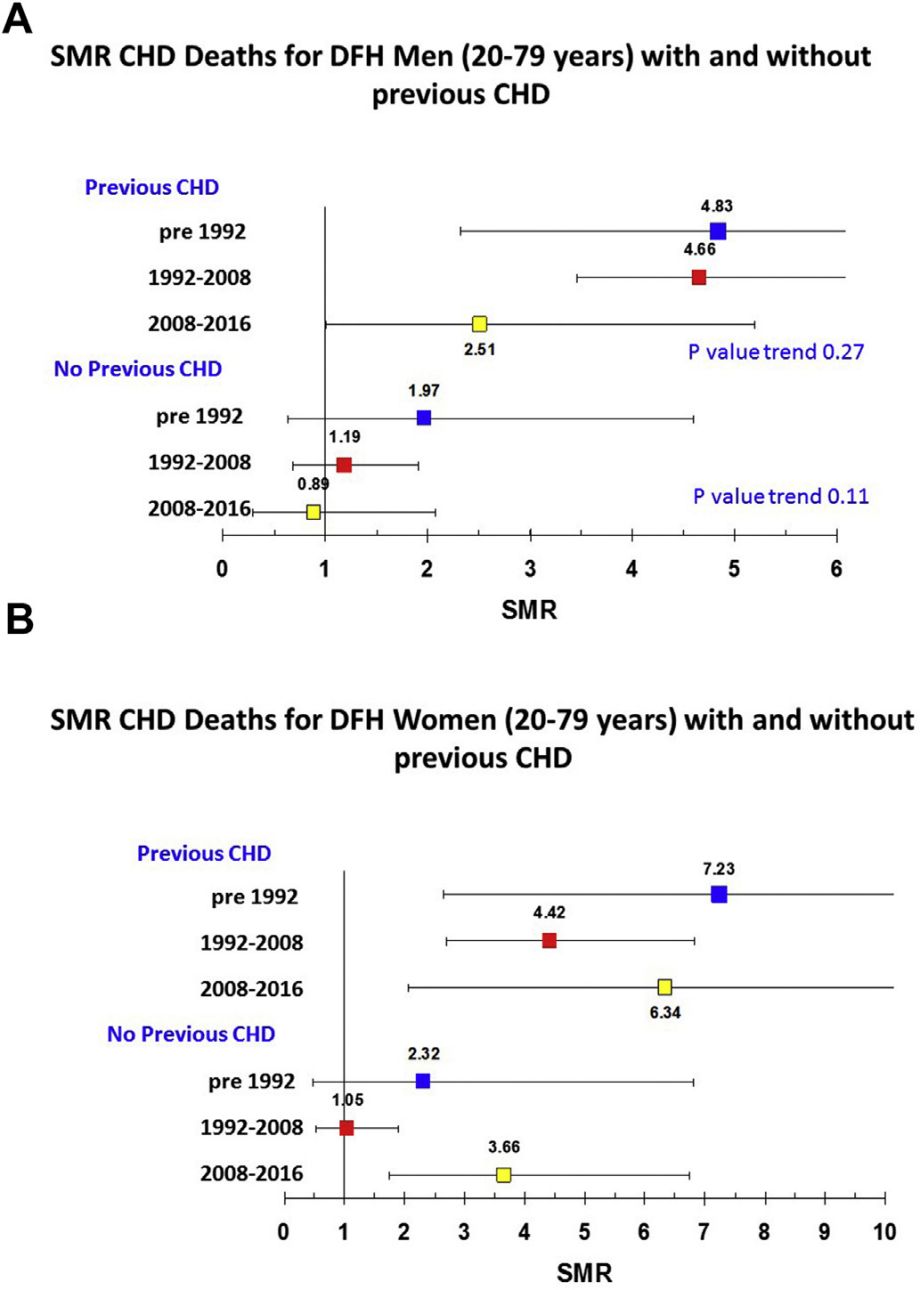
SMR CHD deaths for DFH patients age 20–79 years with and without previous CHD. (A) In 940 DFH men, (B) in 963 DFH women.

Since we now know that the majority of DFH patients have a detectable monogenic cause for their FH phenotype, while the majority of PFH patients have a polygenic cause [15], the remainder of the analysis are restricted to only the DFH patients, in order to obtain estimates of the CHD mortality in treated monogenic FH patients. Also, because our previous analysis showed higher CHD SMR in those with existing CHD on registration [9,16], data are shown in those with CHD at registration (i.e. where statin treatment is secondary prevention) and those with no CHD at registration (i.e. primary prevention). Data are presented in Table 4 and Fig. 1A and B, with a breakdown of SMR by age group in Supplementary Table 4. In treated DFH patients with previous CHD the CHD SMR was significantly elevated at all time periods but in men fell from a 4.83-fold excess (2.32–8.89) pre-1992 to 4.66 (3.46–6.14) in 1992–2008 and 2.51 (1.01–5.17) post 2008, representing a 48% fall overall. By contrast, in women these values were 7.23 (2.65–15.73), 4.42 (2.70–6.82) and 6.34 (2.06–14.81)). In treated DFH men with no previous CHD the excess coronary mortality fell over the three time periods (55% overall), and was not significantly elevated post-2008 (0.89 (0.29–2.08), but in women it remained significantly elevated (post-2008 3.65 (1.75–6.72)). These later values need to be interpreted with caution because of the small number of person years follow up, and low number of events, for example in the last time period for primary prevention in women there were 5 events observed and less than 1 expected, and in secondary prevention there were 10 events observed compared to less than 3 expected.

**Table 4:** Observed and expected CHD deaths by time period in DFH males and females (all ages) with and without CHD at registration.

	Total Pyrs	From 1 January 1980 to 31 December 1991					Total Pyrs	From 1 January 1992 to December 2008				
		Observed	Expected	SMR	95% CI	*p*-value		Observed	Expected	SMR	95% CI	*p*-value
**Male patients**												
CHD	616	10	2.07	483	(232, 589)	0.0001	2940	50	10.7	466	(346, 614)	<0.0001
No CHD	1444	5	2.53	197	(64, 460)	023	8557	17	14.28	119	(69, 191)	0.54
**Female patients**												
CHD	417	6	0.83	723	(265, 1573)	0.0004	1948	20	4.53	442	(270, 682)	<0.0001
No CHD	1410	3	1.29	233	(48, 681)	0.28	9832	11	10.4	105	(53, 189)	0.94

	Total Pyrs	From 1 January 2009 to 31 December 2015					*p*-value trend
		Observed	Expected	SMR	95% CI	*p*-value	
**Male patients**							
CHD	975	7	2.79	251	(101, 517)	0.035	0.11
No CHD	3823	5	5.6	89	(29, 208)	0.99	0.27
**Female patients**							
CHD	535	4	0.77	516	(141, 1323)	0.016	0.50
No CHD	4127	10	2.74	366	(165, 672)	0.001	0.16

Because the SMR calculation is based on a comparison of the mortality rate in FH patients versus the general population, the changes in SMR over time could be influenced by changes in CHD mortality rates in the general population. To examine this we obtained estimates of the CHD mortality rates for men and women in England and Wales, which show that in the general population CHD mortality fell by 21% in males and by 25% in females over the period examined (Supplementary Fig. 2). By contrast, mortality rates in treated DFH patients fell by 60% in males but only by 25% in females. This suggests that the high SMR post-2008 in women with FH is not explained by a disproportionally large fall in the CHD rate in the general population.

Finally we estimated the SMR for different types of cancer, to examine whether the previously reported lower risk for cancer mortality in treated FH patients on the register is maintained in extended follow-up. As shown in Table 4 post 1991, the risk of fatal cancer (17,317 person years exposure) was significantly lower than in the general population (0.70 (0.59–0.83)), mainly due to lower rates of respiratory and lymphatic cancer deaths.

## 4 Discussion

The main new finding of this follow up analysis of the Simon Broome FH registry participants, with over 67,000 person-years and 585 deaths, including 252 from CHD, is that whilst the risk of fatal coronary disease has continued to fall with treatment in men with FH over the three time-periods examined, there has not been a corresponding reduction among women. In DFH men with pre-existing CHD, there was a progressive 48% decrease in CHD SMR from the pre-statin period before 1991, compared to the latest period examined between 2009 and 16. By comparison, in the corresponding group of women with DFH, coronary mortality was similar before and after the introduction of statins. While this could be an artefact due to the relatively small number of events and person years follow up in this later period, it is striking that a similar pattern is observed in both the primary and secondary prevention analyses. This raises the question of whether these DFH women are being treated as rigorously as their male counterparts. For example statin treatment may not be initiated as early as in men, or with statins as potent or in doses to achieve as low therapeutic LDL targets. However, while the overall death rate in this group of treated DFH patients is 20.1 per 1000 person years (315/15674), the CHD death rate is only 9.5/1000 person years (149/15674). This means that, in this group of patients, CHD is the cause of death in only 44% of cases, again supporting the utility of statin treatment in preventing CHD in patients with FH.

Those subjects who were recruited before 1992 were not treated with statins at registration, thus allowing us to estimate the FH CHD SMR mortality without statin treatment. Also post 1992, the statins available at the time of registration were low potency, or, if available, high potency statins were often not prescribed because treatment targets at that time were relatively conservative, in part reflecting initial uncertainty about potential long-term safety. This allows us to estimate changes in CHD SMR in the “low” statin era, compared to that when high intensity statins became available. At registration 42.7% of DFH and 46.2% of PFH patients were on statin treatment and showed a similar (*p*=0.24) 17.5% and 19.5% reduction of LDL-C levels from untreated values. We do not have follow up lipid level data in the Simon Broome registrants, but the inference we have made is that the FH patients on the register have, over time, had their statin therapy up-titrated in line with statin availability and with regard to changing CHD guidelines. Data on current treatment of FH patients comes from the UK 2010 National FH Survey data [21], where 86% of patients were reported to be treated with either atorvastatin or rosuvastatin, and 40% were also on ezetimibe. Mean (SD) untreated LDL-C was 6.44 (1.77) mmol/l, and by the third clinic visit this had been lowered to a mean of 3.60 (1.48) mmol/l, representing an overall median reduction of 47% (IQR 28%–59%) from baseline (Supplementary Fig. 3). and it is reasonable to assume that the Register patients would be similarly treated. However, in the 2010 survey, while 52% of men with CHD achieved a 50% or more reduction in baseline LDL-C only 47% of women with CHD reached this target (p = 0.04), supporting the view that women with CHD are being less effectively treated than men (Supplementary Fig. 4). Several reports have been published recently indicating that women are undertreated with statins [22,23]. In the CASCADE-FH study from the US, compared to men with FH, women with FH were 40% less likely to recive a statin, 32% less likely to achieve the recommended target LDL-C and 21% less likely to achieve 50% LDL-C lowering [22]. Furthermore, in a recent UK general population study, overall women were 24% less likely to initiate or maintain statin therapy compared to men [23].

Using the enlarged data set, we were able to confirm several of our earlier findings. As reported [19], in those with a clinical diagnosis of Definite FH, the SMR was 35% higher in the post 1991 period compared to those with Possible FH. Overall, CHD risk was independently related to age, sex, smoking, previous CHD and total cholesterol, but after adjustment for these variables the higher CHD risk in DFH compared to PFH patients was no longer statistically significant. Since we know that up to 80% of those with clinical DFH will carry an FH-causing mutation [13,14] while probably 80% of those with clinical PFH have a polygenic aetiology of their hyperlipidaemia [15,16], this confirms reports that carriage of an FH mutation is associated with greater CHD risk [4]. This higher risk is supported by the observation that, even though LDL-C levels were similar, compared to those with polygenic hypercholesterolaemia, those with an FH-causing mutation have greater carotid intimal-medial thickening and coronary calcification [24]. As previously reported [8,9], the CHD SMR in younger FH patients was extremely high, and Supplementary Table 4 shows that although the absolute event rate in patients aged 20–39 was low at all time periods, the CHD SMR in the DFH men and women with no previous CHD fell pre 1992 from 3750 (773–10959) to 1153 (238–3372) in 1992–2008, but was 5601 (678–20233) post 2008.

Since many recent guidelines for the management of FH [1] recommend use of the DLCN score for the diagnosis of FH, we compared the CHD SMR of those with Simon Broome DFH and PFH with the DLCN score equivalent groups (Score >8 and 5–8 respectively). The data shows that these two diagnostic criteria are equally good at distinguishing those with clinical FH who have a higher or lower CHD risk.

We were able to confirm our earlier report [18] that in DFH patients who did not have CHD at registration, the CHD SMR in the post-statin period is not greater than the general population, supporting the significant clinical utility of identifying subjects with FH before they have developed CHD and ensuring they receive intensive lipid-lowering therapy. We were also able to confirm [18] the findings that this cohort of FH patients continue to show lower mortality rates of particularly smoking-related cancers, due most likely to the lower rate of smoking and other positive life-style changes recommended for these patents during their ongoing care by lipid clinics. Data from the Health Survey for England (https://digital.nhs.uk), show that in 1993, 28% of men and 26% of women in the UK self-reported as smokers, but by 2016 these figures had fallen to 20% and 16% respectively. This compares with a lower smoking prevalence at registration of 16.8% in the FH men and 19.8% in women, while in the 2010 FH audit [21] the prevalence was 14%. The lower cancer mortality seen may also, in part, be because of earlier detection and treatment of cancer among patients undergoing regular medical surveillance, resulting in a better prognosis. We cannot, however, entirely exclude the possibility that statins have anti-cancer activity, although a meta-analysis of 27 randomised trials of statin therapy over a relatively short period of about five years showed no difference in the incidence or mortality from cancers [25].

### 4.1 Strengths and limitations

There are a number of strengths of the current study. Follow-up of the cohort is essentially complete, with an observation period now including more than 67,000 patient years, with an increase in the duration of follow up of 45% since the 2008 analysis. Our study is one of the largest long-term data sets available with only that in Holland being larger. The SMRs are relevant for estimating the mortality in untreated and treated FH patients in the UK and can robustly inform health economic modelling, as has been done recently [25,26]. We have no information on deaths since 2016 which might be used to strengthen our data. The Register only has information on registration levels of lipids and historical on-treatment lipids, but does not have recent on-treatment lipid data so we cannot say with certainty what current treatment or LDL-C levels are in any of the subjects. At the time of the UK 2010 National Survey, most FH patients were reported to be treated with either atorvastatin or rosuvastatin, 40% were also on ezetimibe, and it is reasonable to assume that the Register patients would be similarly treated. The major limitations are due to the relatively small number of deaths occurring in the latest period between 2008 and 2016. In the whole cohort this includes more than 17,000 person years and 54 CHD deaths, but in the DFH men with CHD this is 975 person years and 7 events observed (2.8 expected), while in the DFH women this is 535 person years with 4 events observed (0.8 expected). Because of this, the confidence intervals are large and the point estimates need to be interpreted cautiously.

In conclusion, these data confirm the benefit of statin treatment in reducing CHD mortality in patients with a clinical diagnosis of FH, but suggest that, despite recent European [1] and UK guidelines recommending the use of potent statins to lower LDL-C, FH patients with pre-existing CHD, and a proportion of women with FH, may not be treated adequately. Because of their high CHD risk, patients with FH cannot be recruited into a randomised-controlled trial of placebo versus lipid lowering therapy, but long-term follow-up registers such as used here, and the Familial Hypercholesterolaemia Studies Collaboration [26] can provide valuable data to examine the utility of statin treatment.

## Conflicts of interest

HAWN, NC, PND, BJ, IFWM, HS and MS have served as consultants to pharmaceutical companies marketing lipid-lowering drugs, and have received travel expenses, payment for speaking at meetings and funding for research from some of these companies.

## Financial support

The Simon Broome FH register was previously supported by an unrestricted educational grant from Pfizer Ltd, and has previously received support from Astra Zeneca and Schering-Plough Ltd. HAWN and SEH would like to acknowledge grants RG3008 and RG008/08 from the British Heart Foundation, BJ the support of an NIHR Clinical Lectureship and SEH the support of the UCLH NIHR BRC. The sponsors had no involvement in any aspect of the study or publication.

## Appendix A Supplementary data

Supplementary data v5 Supplementary data v5
