# Supplementary material for: Coronary heart disease mortality in treated familial hypercholesterolaemia: Update of the UK Simon Broome FH register
Source: Atherosclerosis. 2018 Jul;274:41–6. doi: 10.1016/j.atherosclerosis.2018.04.040 (PMC6013645; doi:10.1016/j.atherosclerosis.2018.04.040)
Supplement: Supplementary data v5 [file mmc1.docx]

**Supplementary data**

**Supplementary Methods**

Recruitment to the Simon Broome Register of patients with heterozygous familial hypercholesterolamia began in 1980 and continues. Homozygous patients were excluded. The 21 participating clinics registered patients referred to them by either general practitioners or hospital specialists.

A **Simon Broome diagnosis** of definite familial hypercholesterolaemia in adults was defined as described [9] with a pre-treatment or on treatment total cholesterol above 7.5 mmol/l (or, when available, a low density lipoprotein cholesterol above 4.9 mmol/l) together with the presence of tendon xanthomas either in the patient or in a parent, child, grandparent, sibling, uncle or aunt. Possible familial hypercholesterolaemia was defined using the same cholesterol criteria together with either a family history of myocardial infarction before age 50 in 2^nd^ degree relative or before age 60 in 1^st^ degree relative or, alternatively, a family history of raised total cholesterol concentration above 7.5 mmol/l in 1^st^ or 2^nd^ degree relative. The original definition was subsequently amended so that definite familial hypercholesterolaemia could be defined by the elevated cholesterol concentration and evidence of an *LDLR*, an *APOB* or *PCSK9* mutation, but for this analysis the clinical criteria were used exclusively.

The **Dutch Lipid Clinic Network** scoring criteria for FH diagnostic criteria for index FH individuals is shown below. A ‘definite’ diagnosis requires more than 8 points, a ‘probable’ diagnosis requires 6-8 points, a ‘possible’ diagnosis requires 3-5 points, and below 3 points is not FH [10].

| **Dutch Lipid Clinic Network Criteria** | | **points** |
| --- | --- | --- |
| Family history | 1^st^-degree relative with known premature ( men <55 years and women < 60 years) coronary and vascular disease or 1^st^-degree relative with known LDL-C above the 95^th^ p%ile | 1 |
|  | 1^st^-degree relative with tendon xanthomata and/or arcus cornealis, or children aged less than 18 years with LDL-C above the 95^th^ %ile | 2 |
| Clinical history | Patient with premature coronary artery disease | 2 |
|  | Patient with premature cerebral or peripheral vascular disease | 1 |
| Physical examination | Tendon xanthomata | 6 |
|  | Arcus cornealis prior to age 45 years | 4 |
| Cholesterol levels | LDL-C >=8.5 | 8 |
|  | LDL-C 6.5-8.4 | 5 |
|  | LDL-C 5.0-6.4 | 3 |
|  | LDL-C 4.0-4.9 | 1 |
| DNA analysis | Functional mutation in the *LDLR* (*APOB/PCSK9*) gene | 8 |

**Haralambos et al Atherosclerosis 2015**

Physicians and clinics that have participated in the Simon Broome Register at any time from 1980 onwards:

K. Arnsten, S. Flemming (Royal Cornwall Hospital, Truro), D.J. Betteridge (University College Hospital, London), P.N. Durrington (Manchester Royal Infirmary), R.S. Elkeles (St Mary’s Hospital, London), R.M. Finnie (St John’s Hospital, Livingston), D.J. Galton (St Bartholomew’s Hospital, London), R. Hillson (Hillingdon Hospital, Uxbridge, and Mount Vernon Hospital, Northwood), E.A. Hughes (Sandwell District Hospital, West Bromwich), M.F. Laker (Royal Victoria Infirmary, Newcastle-Upon-Tyne), B. Lewis, A.S. Wierzbicki (St Thomas’s Hospital, London), R. Lorimor (Glasgow Royal Infirmary), J.I. Mann (John Radcliffe Hospital, Oxford), D.R. Matthews, H.A.W. Neil (Churchill Hospital, Oxford), J.P. Miller (University Hospital of South Manchester, J.P.D. Reckless (Royal United Hospital, Bath), L.N. Sandle (Trafford General Hospital, Manchester), M. Seed (Charing Cross Hospital, London, and King Edward VII Hospital), K.G. Taylor (City Hospital, Birmingham), G.R. Thompson, R Naoumova (Hammersmith Hospital, London), R. West (St George’s Hospital, London).

**Supplementary Figures**

**Supplementary Figure 1. SMR for CHD Deaths for Simon Broome DFH vs DLCN Score >8 and Simon Broome PFH vs DLCN <8**

Not all subjects had the necessary data to determine DLCN score. SMR in DFH and PFH estimated only in those with DLCN score. SMRs are SB DFH (n=1903) = 2.53 (2.14-2.97), DLCN >8 (n=1297) = 2.53 (2.07-2.05) and in SB PFH (n=1650) = (1.85 (1.51-2.24) and DLCN <8 (n=1632) = 1.65 (1.30-2.06).


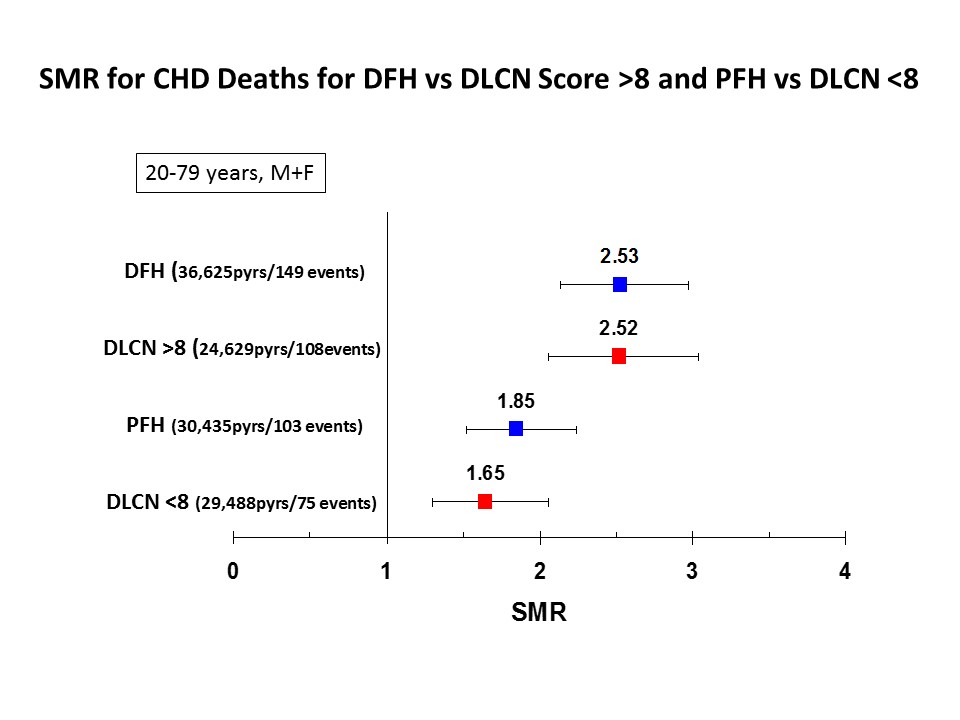


**Supplementary Figure 2** **CHD Mortality rates in the men and women from the population of England and Wales and in DFH patients by time period.**


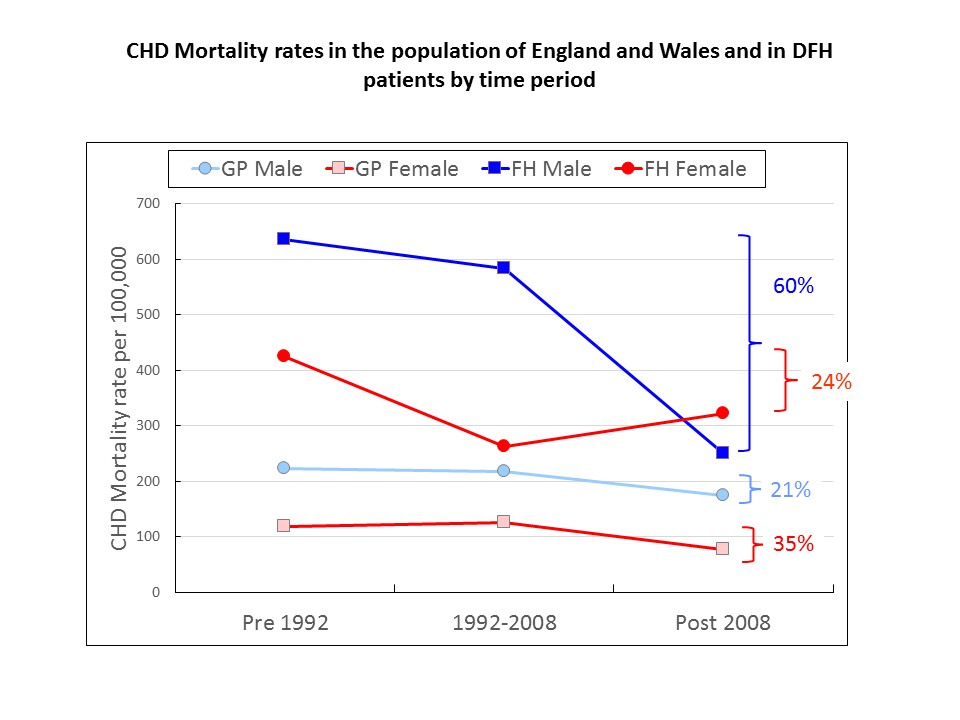


GP Male/GP Female (GP = General Practice). The CHD mortality data was obtained from the Health Survey of England and Wales website. [**https://www.ons.gov.uk/peoplepopulationandcommunity/birthsdeathsandmarriages/deaths/datasets/the21stcenturymortalityfilesdeathsdataset**](https://www.ons.gov.uk/peoplepopulationandcommunity/birthsdeathsandmarriages/deaths/datasets/the21stcenturymortalityfilesdeathsdataset)

**Supplementary Figure 3. LDL-C levels pre- treatment and at third clinic visit in adults on statin therapy from the UK 2010 National FH Survey (Seed et al (22).**

**
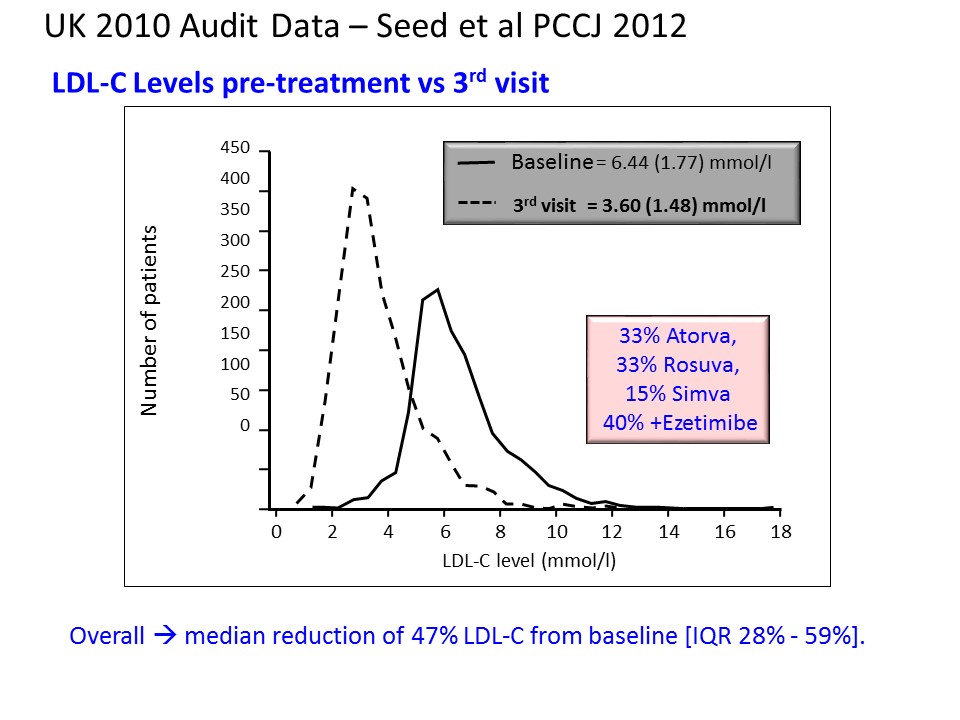
**

**Supplementary Figure 4. Proportion statin-treated FH patients achieving 50% LDL-C lowering by third clinic visit in adults from the UK 2010 National FH Survey (Seed et al (22).**

**
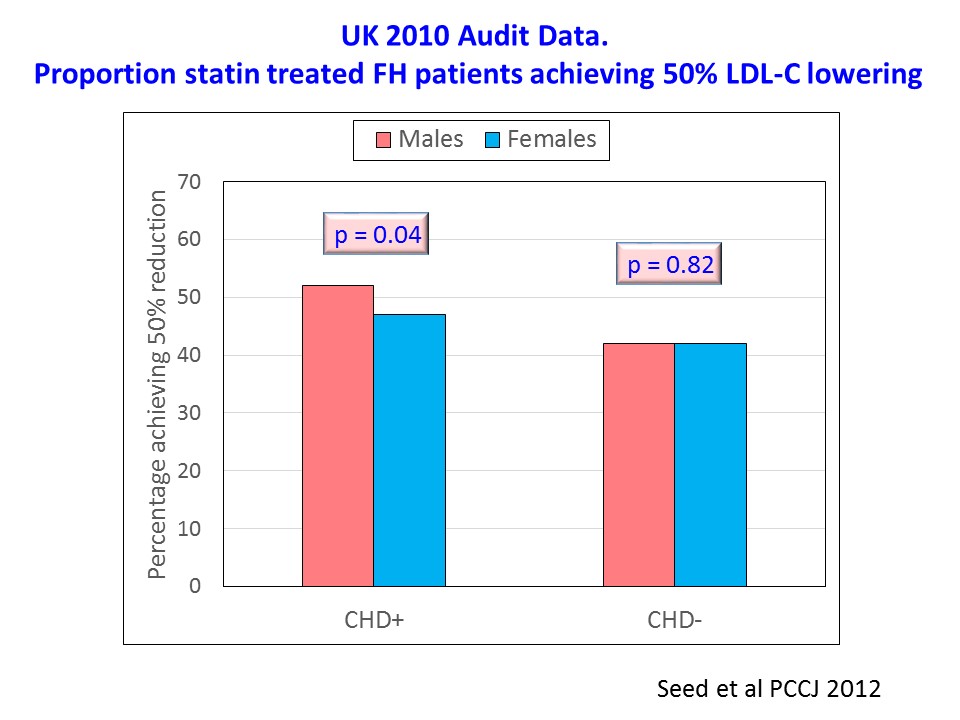
**

**SupplementaryTable 1 Characteristics (mean +SD) in those with Simon Broome DFH or PFH**

**.**

|  | **DFH**  **N=1903** | **PFH**  **N=1650** | **P value** |
| --- | --- | --- | --- |
| Median age [IQR] | 42.8 [30.5 -55.2] | 47.7 [35.7-57.9] | <0.001 |
| Number (%age) Male | 940 (49.4) | 784 (47.5) | 0.26 |
| Previous MI | 188 (10.0%, n=1875) | 180 (11.0%, n=1633) | 0.34 |
| Current/past angina | 337 (17.8, N=1891) | 277 (16.9, N=1643) | 0.45 |
| Diagnosed CHD | 445 (23.4%, N=1903) | 363 (22.0%, N=1650) | 0.33 |
| Previous stroke | 13 (0.7%, N=1880) | 32 (2.0%, N=1630) | 0.001 |
| Diagnosed diabetes | 16 (0.9%, N=1884) | 29 (1.8%, N=1633) | 0.015 |
| Current cigarette smoker | 312 (16.8%, N=1853) | 315 (19.8%, N=1594) | 0.026 |
| SBP (mmHg) | 130.3 (20.8, N=1784) | 134.5 (20.7, N=1556) | <0.001 |
| DBP (mmHg) | 78.9 (11.3, N=1780) | 81.4 (11.4, N=1556) | <0.001 |
| BMI (kg/m^2^)^1^ | 24.7 (4.3, N=1707) | 25.5 (4.6, N=1405) | <0.001 |
| Total cholesterol (mmol/l) | 8.25 (2.19, N=1835) | 7.57 (1.82, N=1601) | <0.001 |
| Triglyceride (mmol/l)^2^ | 1.32 ( 0.9-1.9 , N=1787) | 1.58 (1-2.3 , N=1563) | <0.001 |
| HDL-C (mmol/l) | 1.28 (0.4, N=1578) | 1.35 (0.43, N=1348) | <0.001 |
| LDL-C (mmol/l) | 6.61 (2.17, N=1533) | 5.60 (1.75, N=1283) | <0.001 |

Denominators differ because of missing measurements.

^1^ A number of individuals excluded because of incorrect weight data

^2^ Geom. mean [IQR].

**Supplementary Table 2 - Clinical characteristics at registration by Sex**

_____________________________________________________________________________________

Men Women

(n=1724) (n=1829)

_____________________________________________________________________________________________________________________

Median age (inter-quartile range) 42.3 (32.2, 52.3) 49.4 (33.3, 59.8).

Previous myocardial infarction 249 (14.6%, n=1707) 119 (6.6%, n=1812)

Current or past angina 331 (19.5%, n=1696) 283 (15.6%, n=1809)

Diagnosed coronary heart disease 466 (27.0%, n=1724) 342 (18.7%, n=1829)

Previous stroke 21 (1.2%, n=1704) 24 (1.3%, n=1814)

Diag(osed diabetes 25 (1.5%, n=1704) 20 (1.1%, n=1813)

Current cigarette smoker 274 (16.4%, n=1668) 353 (19.8%, n=1779)

Systolic blood pressure (mm Hg) 130.3 (18.9, n=1611) 134.0 (22.4, n=1729)

Diastolic blood pressure (mm Hg) 79.6 (11.2, n=1610) 80.4 (11.6, n=1726)

Body mass index (kg/m^2^) 25.2 (4.0, n=1494) 24.9 (4.8, n=1608)

Total cholesterol (mmol/l) 7.8 (1.9, n=1665) 8.1 (2.2, n=1771)

Triglycerides (mmol/l, geometric mean & IQR) 1.5 (1.0, 2.3, n=1622) 1.3 (0.9, 1.9, n=1728)

High density lipoprotein cholesterol (mmol/l) 1.2 (0.3, n=1410) 1.4 (0.4, n=1516)

Low density lipoprotein cholesterol^†^ (mmol/l) 6.0 (2.0, n=1343) 6.3 (2.1, n=1473)

_____________________________________________________________________________

Results are presented as mean (SD) unless otherwise stated

Diagnosed CHD defined as a previous myocardial infarction, or angina, or coronary artery bypass graft, or angioplasty

† LDL cholesterol concentrations were calculated according to Friedewald et al. (20)

**Supplementary Table 3 Characteristics (mean +SD) in those with DCLN score below and above 8.**

|  | **DCLN>8**  **N=1297** | **DCLN<8**  **N=1632** | **P value** |
| --- | --- | --- | --- |
| Median age [IQR] | 46.4 [36.2-57.0] | 44.0 [28.7-56.4] | <0.001 |
| Number (%age) Male | 621 (47.9%, N=1297) | 775 (47.5%, N=1632) | 0.83 |
| Previous MI | 184 (14.4%. N=1282) | 113 (7.0%, N=1614) | <0.001 |
| Current/past angina | 269 (20.9%, N=1285) | 225 (13.8%, N=1629) | <0.001 |
| Diagnosed CHD | 372 (28.7%, N=372) | 281 (17.2%, N=281) | <0.001 |
| Previous stroke | 11 (0.9%, N=1288) | 21 (1.3%, N=1628) | 0.26 |
| Diagnosed diabetes | 8 (0.6%, N=1292) | 18 (1.1%, N=1630) | 0.17 |
| Current cigarette smoker | 246 (19.3%, N=246) | 272 (17.2%, N=1584) | 0.14 |
| SBP (mmHg) | 133.3 (21.2, N=1275) | 131.2 (20.7, N=1528) | 0.007 |
| DBP (mmHg) | 80.2 (11.5, N=1273) | 79.7 (11.5, N=1527) | 0.26 |
| BMI (kg/m^2^)^1^ | 25.3 (4.0, N=1175) | 25.1 (4.8, N=1429) | 0.28 |
| Total cholesterol (mmol/l) | 8.74 (2.28, N=1289) | 7.19 (1.51, N=1621) | <0.001 |
| Triglyceride, (mmol/l)^2^ | 1.35 (0.9-1.9) ,N=1285 | 1.41 (0.9-2.1), N=1611 | 0.049 |
| HDL-C (mmol/l) | 1.29 (0.37, N=1261) | 1.33 (0.38, N=1579) | <0.001 |
| LDL-C (mmol/l) | 7.15 (2.23, N=1254) | 5.35 (1.47, N=1562) | <0.001 |

1 Some individuals excluded because of incorrect weight data

2 Geom. mean [IQR].

**Jackie does JJA**

Supplementary Table 4 – Observed and expected deaths from CHD by age group and time period for DFH patients with and without known CHD at registration

|  | **1 January 1980 to 31 December 1991** | | | | | | | **1 January 1992 to 31 December 2008** | | | | | | |
| --- | --- | --- | --- | --- | --- | --- | --- | --- | --- | --- | --- | --- | --- | --- |
| Attained age (years) | Person years observation | Observed | Expected | SMR | 95% CI | **p-value** | **Rate/ 100000** | **Person years observation** | **Observed** | **Expected** | **SMR** | **95%CI** | **p-value** | **Rate/ 100000** |
| Primary Prevention | |  | | | | | |  | | | | | | |
| 20-39 | 2031 | 3 | 0.08 | 3750 | (773, 10959) | <0.001 | 148 | 8227 | 3 | 0.26 | 1153 | (238, 3372) | <0.01 | 37 |
| 40-59 | 2181 | 8 | 2.34 | 342 | (148, 674) | <0.01 | 367 | 13123 | 13 | 9.19 | 141 | (75, 242) | 0.28 | 99 |
| 60-79 | 686 | 1 | 3.63 | 27 | (1, 153) | 0.25 | 146 | 8219 | 29 | 34.33 | 84 | (57, 121) | 0.41 | 353 |
| 0-79 | 4898 | 12 | 6.05 | 198 | (102, 346) | 0.04 | 212 | 29569 | 45 | 43.78 | 103 | (75, 138) | 0.89 | 145 |
| **Secondary Prevention** | |  | | | | | |  | | | | | | |
| 20-39 | 178 | 5 | 0.01 | 50000 | (16235, 116683) | <0.0001 | 2816 | 229 | 1 | 0 |  |  | 0 | 436 |
| 40-59 | 1016 | 9 | 1.58 | 570 | (260, 1081) | <0.0001 | 886 | 3419 | 34 | 3.83 | 888 | (615, 1241) | <0.0001 | 995 |
| 60-79 | 539 | 11 | 3.24 | 340 | (169, 607) | <0.001 | 2038 | 4509 | 73 | 24.01 | 304 | (238,382) | <0.0001 | 1619 |
| 0-79 | 1733 | 25 | 4.83 | 515 | (335, 764) | <0.0001 | 1442 | 8157 | 108 | 27.84 | 388 | (318, 468) | <0.0001 | 1324 |

|  | **1 January 2009 to 31 December 2015** | | | | | | |
| --- | --- | --- | --- | --- | --- | --- | --- |
| Attained age (years) | Person years observation | Observed | Expected | SMR | 95% CI | **p-value** | **Rate/ 100000** |
| Primary Prevention | |  | | | | | |
| 20-39 | 1405.9 | 2 | 0.04 | 5601 | (678,20233) | 0.001 | 142 |
| 40-59 | 3434.7 | 0 | 1.23 | 0 | (0,300) | 0.58 | 0 |
| 60-79 | 3109 | 13 | 7.08 | 184 | (98,314) | 0.06 | 418 |
| 0-79 | 7949.7 | 15 | 8.34 | 180 | (101, 297) | 0.05 | 189 |
| **Secondary Prevention** | |  | | | | | |
| 20-39 | 5 | 0 | 0 | 0 | - | - | - |
| 40-59 | 336.5 | 1 | 0.20 | 499 | (13, 2782) | <0.0001 | 886 |
| 60-79 | 1168.6 | 11 | 3.38 | 325 | (162, 582) | <0.001 | 2038 |
| 0-79 | 1510.1 | 12 | 3.58 | 335 | (173, 585) | <0.0001 | 1442 |

**Survivor effect seems to be wearing off as wells!**

**Supplementary** **Table 5 – Observed and expected deaths for all cancers and site-specific cancers by time period**

|  |  | **From 1 January 1980 to 31 December 1991 Person-years exposure = 6627years** | | | | | **From 1 January 1992 to 31 December 2015 Person-years exposure = 17317 years** | | | | |
| --- | --- | --- | --- | --- | --- | --- | --- | --- | --- | --- | --- |
| **Specified site** | **ICD 9 codes** | **Observed** | **Expected** | **SMR** | **95% CI** | **p-value** | **Observed** | **Expected** | **SMR** | **95% CI** | **p-value** |
| **All cancers** | 1400-2089 | 14 | 14.71 | 95 | (52, 160) | 0.99 | 147 | 208.86 | 70.4 | (59.83) | <0.0001 |
| **Lip, oral cavity & pharynx** | 1400-1499 | 1 | 0.21 | 466 | (12, 2595) | 0.39 | 2 | 3.03 | 66.0 | (8,239) | 0.83 |
| **Digestive organs & peritoneum** | 1500-1599 | 3 | 3.74 | 80 | (17,234) | 0.97 | 42 | 57.85 | 72.6 | (52,98) | 0.036 |
| **Respiratory & intrathoracic organs** | 1600-1699 | 1 | 3.91 | 26 | (1, 143) | 0.20 | 20 | 52.98 | 37.8 | (23,58) | <0.0001 |
| **Bone, connective tissue, skin & breast** | 1700-1759 | 1 | 2.27 | 44 | (1,246) | 0.68 | 28 | 22.67 | 123.5 | (82,178) | 0.31 |
| **Genitorurinary** | 1790-1899 | 2 | 2.10 | 95 | (12, 344) | 1 | 25 | 31.54 | 79.3 | (51,117) | 0.28 |
| **Other solid cancers** | 1900-1999 | 3 | 1.48 | 203 | (42, 594) | 0.37 | 18 | 13.49 | 133.4 | (79,211) | 0.28 |
| **Lymphatic & haemopoetic tissue** | 2000-2089 | 3 | 1.00 | 300 | (62, 878) | 0.16 | 11 | 25.48 | 43.2 | (22,77) | 0.002 |

.
